# Reassessing the Necessity of Group and Save Testing in Ambulatory Orthopedic Peripheral Limb Trauma Surgery

**DOI:** 10.7759/cureus.96678

**Published:** 2025-11-12

**Authors:** Max Moss, Conor Boylan, Success Anyanwu, Tom Havenhand

**Affiliations:** 1 Trauma and Orthopaedics, Royal Bolton Hospital, Bolton, GBR; 2 Plastic Surgery, Alder Hey Children's Hospital, Liverpool, GBR; 3 Orthopaedics and Trauma, Pennine Acute NHS Foundation Trust, Manchester, GBR

**Keywords:** k-wire, lower limb trauma, open reduction internal fixation, preoperative group and save, upper limb surgery

## Abstract

Background: Preoperative group and save (G&S) testing is routinely ordered for patients undergoing ambulatory peripheral limb orthopedic trauma procedures due to anticipated perioperative blood loss. However, national and local guidance on this protocol is often ambiguous, leading to potentially unnecessary testing with associated economic and environmental costs.​ This study aimed to determine the incidence of red cell transfusion in adult patients undergoing ambulatory peripheral limb trauma surgery, quantify the prevalence of preoperative G&S testing, and identify patient and procedural factors associated with the ordering of G&S tests.

Methods: This retrospective cohort study included all adult patients undergoing ambulatory orthopedic procedures (open reduction internal fixation and K-wire surgeries) distal to the shoulder and knee joints at a single NHS trust over a 12-month period (July 2024-July 2025). Data collected included patient demographics, incidence of G&S testing, and perioperative red cell transfusion rates. Statistical analysis used univariable and multivariable models to identify predictors of G&S testing.

Results: A total of 459 patients (mean age 50.4+/-19.5 years), 157 (34.3%) had a G&S test performed, resulting in a total of 315 samples. The ankle was the site with the most frequent G&S testing (n=43, 62.6% of ankle procedures). American Society of Anaesthesiologists (ASA) grade 2 was the most frequent ASA grade (n=227, 49.5%). Mean preoperative hemoglobin was 132.1±18.3 g/L. One patient (0.21%) received a preoperative transfusion for pre-existing anemia.

No patients received a postoperative red cell transfusion. Multivariable logistic regression found no clinical significance between demographics and the likelihood of G&S testing. Poisson regression analysis found that upper limb procedures were associated with significantly fewer tests (incidence rate ratio (IRR)=0.20, p=0.009), while higher ASA grade (p=0.024) and procedures involving the humerus/shoulder (IRR=3.0, p=0.037) were associated with increased G&S testing.

Conclusion: This study demonstrates that routine preoperative G&S testing is not clinically required for many patients undergoing ambulatory peripheral limb orthopedic trauma surgery. Current testing practices, influenced by procedural site and patient comorbidity (ASA grade), contribute to a significant and unnecessary economic and environmental cost. Implementing a selective, risk-based institutional protocol is necessary to align practice with evidence, improve cost-effectiveness, and enhance the sustainability of orthopedic surgical care.

## Introduction

Ambulatory peripheral limb trauma constitutes a substantial portion of orthopedic trauma presentations in the United Kingdom [1]. Recent multicenter analyses show that upper and lower limb fractures remain the dominant driver of orthopedic trauma workload, and absolute caseloads are projected to rise with the increasing burden of fragility fractures in an aging population [2-5].

Despite the generally low expected blood loss, preoperative group and save (G&S) testing is requested routinely across many orthopedic units. This habitual practice may be driven by a lack of clear national or local guidance, particularly for lower-risk surgical cohorts; the National Institute for Health and Care Excellence (NICE) provides no specific recommendations for G&S testing in acute fractures [6]. Unlike major arthroplasty, femoral fracture fixation, or pelvic trauma, where transfusion risk is well-documented, specific protocols for peripheral limb trauma are often nonexistent or vague [7,8].

The rationale for routine G&S testing is based on the rare but potential need for urgent perioperative transfusion. However, several studies across various low-blood-loss surgeries have demonstrated exceedingly low or zero transfusion rates [9,10]. In the specific context of ambulatory peripheral limb orthopedic trauma, there is a clear deficiency of high-quality data to justify routine blood testing, with only one recent study demonstrating minimal risk of transfusion [11]. Without robust evidence, the current approach risks over-investigating patients who possess a high intrinsic physiological reserve and low procedural risk, while diverting resources from patients who truly require them.

Unnecessary preoperative testing carries direct financial as well as indirect costs, such as disrupted operating list flow, patient anxiety, and increased environmental burden [12]. In line with the NHS goal of achieving a net-zero health service, every unit of unnecessary testing, including the consumables, power consumption, and transportation required for G&S sampling, represents a failure to adopt a more sustainable surgical practice. Therefore, determining the actual incidence of transfusion is critical for developing a data-driven, cost-effective, and environmentally conscious protocol.

This study aimed to determine the incidence of perioperative red cell transfusion in patients undergoing ambulatory peripheral limb orthopedic trauma surgery, quantify the prevalence of G&S testing in this cohort, and identify patient and procedural factors that influence G&S test ordering. Secondary aims were to evaluate the clinical utility and associated economic and environmental costs of the current routine testing protocol.

## Materials and methods

Study design and setting

This was a retrospective cohort study conducted at a single NHS acute trust. Data was collected during a 12-month period from July 2024 to July 2025. Ambulatory procedures were defined as those performed on planned trauma lists that did not necessitate emergency admission or require a postoperative stay beyond 24 hours. The study was registered as a service evaluation (approval number 5055) and did not require ethical approval as per governance guidelines.

Inclusion criteria

All adult patients (aged ≥16 years) undergoing upper limb trauma surgery and K-wire/ORIF (open reduction and internal fixation) procedures distal to the femur were included. This included fractures of the clavicle, humerus, radius, ulna, hand, patella, tibia, ankle, and foot. Patients were excluded if they: (1) were under 16 years of age, (2) underwent procedures involving simultaneous fixation of the femora or pelvic ring, (3) underwent complex revision surgery, or (4) required intramedullary nailing of long bones.

Data collection and analysis

Data on operation type and date of surgery, along with patient demographics, including age, sex, and American Society of Anesthesiologists (ASA) grade, were collected from the ORMIS (Operating Room Management Information System) and Bluespier clinical management system. The number of G&S tests prior to surgery, data on perioperative transfusion requirements, and preoperative hemoglobin levels were collected from ICE (Integrated Clinical Environment). A hemoglobin concentration of <130 g/L was established as the threshold for anemia as per the British Hematology Society [13]. The primary outcomes evaluated were the incidence of G&S testing (documented orders on ICE) and the incidence of perioperative red cell transfusion.

Descriptive statistics were performed using Microsoft Excel (Version 16.101.3, Microsoft Corporation, Redmond, WA) and used to summarize the demographic data and prevalence of G&S testing. G&S and transfusion rates were calculated as a percentage of the total cohort. Continuous variables were presented as mean ± standard deviation, and categorical variables as counts and percentages. To determine the factors associated with G&S testing, both univariable and multivariable analyses were employed. Multivariable logistic and Poisson regression models were used to determine clinical significance and associated 95% confidence intervals. Covariables, including age, sex, ASA grade, preoperative hemoglobin, and fracture site, were included in the multivariable model. All statistical analysis was performed using R Software (R Foundation for Statistical Computing, Vienna, Austria), with a p-value of <0.05 considered statistically significant.

## Results

Patient demographics, G&S, and transfusion incidence

A total of 459 patients underwent ORIF or K-wire fixation procedures during the study period, with a mean age of 50.4±19.5 years. Of these, 205 (44.7%) patients were male and 254 (55.3%) were female. The most common ASA grade was grade 2, representing 49.5% (n=227) of the cohort. The most common fracture site included was the forearm/wrist/elbow (n=162, 35.3%), followed by the ankle (n=116, 25.3%). Preoperative hemoglobin measurement was available for 385 (83.9%) patients with a mean concentration of 132.1±18.3 g/L.

G&S tests were performed on 157 (34.3%) patients, with a total of 315 samples being taken across the study period. Of these, 215 (68.3%) were taken for lower limb procedures and 100 (31.7%) for upper limb procedures. No postoperative red blood cell (RBC) transfusions were required, and one patient (0.21%) required 2 units of RBCs preoperatively for pre-existing anemia.

Full baseline characteristics and G&S testing are illustrated in Table 1. Further breakdown of G&S testing for each fracture type can be seen in Table 2.

**Table 1:** Baseline characteristics of study sample ASA, American Society of Anaesthesiologists

Characteristics	Cohort (n=459)
Sex, n (%)	
Male	205 (44.7)
Female	254 (55.3)
Age, years, mean ± SD	50.4±19.5
ASA grade, n (%)	
1	120 (26.1)
2	227 (49.5)
3	101 (22.0)
4	11 (2.4)
Fracture type, n (%)	
Hand	46 (10.0)
Forearm/wrist/elbow	162 (35.3)
Humerus/shoulder	84 (18.3)
Tibia/patella	35 (7.6)
Ankle	116 (25.3)
Foot	16 (3.5)
Preoperative hemoglobin (g/L), mean ± SD	132.1±18.3
Group & save status, n (%)	157 (34.3)
Transfusion status, n (%)	1 (0.21)

**Table 2:** G&S testing by fracture site G&S, group and save

Fracture type (n)	G&S performed (%)
Hand (46)	4 (8.7)
Forearm/wrist/elbow (162)	24 (14.8)
Humerus/shoulder (84)	29 (34.5)
Tibia/patella (35)	21 (60)
Ankle (116)	72 (62.1)
Foot (16)	7 (43.8)

Predictors of G&S testing

Univariable analysis was used to assess for associations between baseline characteristics and the likelihood of G&S testing. Statistically significant findings were found between multiple baseline characteristics. Anemia and lower limb fracture type were associated with increased likelihood of G&S testing alongside lower limb or humerus/shoulder fracture sites (Chi-square test, X2=25.84, p<0.001, X2=76.83, p<0.001, X2=92.49, p<0.001). Low preoperative hemoglobin and older age were also significant predictors for G&S testing (Mann-Whitney U test, W=21230, p<0.001, W=20362, p<0.015) as well as higher ASA grade (Jonckheere Terpstra test, T=28652, p<0.001). A full summary of the analysis is given in Table 3.

**Table 3:** Summary of univariable analysis Assessment of the significance of the relationship between baseline characteristics and the likelihood of G&S testing included: (1) Chi-square test, sex, anemia status, upper/lower limb, and fracture area; (2) Mann-Whitney U, age and preoperative hemoglobin; and (3) Jonckheere-Terpstra, ASA grade. A p-value of <0.05 was considered statistically significant. ASA, American Society of Anaesthesiologists

Variable	Statistical test	Statistical value	p-value
Sex	Chi-square	X^2^=1.31	0.253
Age	Mann-Whitney U	W=20,362	0.015
Limb (upper vs. lower)	Chi-square	X^2^=76.83	<0.001
ASA grade	Jonckheere Terpstra	JT=28,652	<0.001
Preop hemoglobin	Mann-Whitney U	W=21,230	<0.001
Fracture type	Chi-square	X^2^=92.49	<0.001
Anemia status	Chi-square	X^2^=25.84	<0.001

When adjusting for confounding variables, no statistical significance was found between baseline characteristics and the binary outcome of whether a G&S test was performed. The same characteristics were entered into a Poisson regression model to assess significance against the number of tests performed in the patients who had G&S testing; this model found several independent predictors of increased G&S testing. Patients undergoing upper limb procedures were less likely to have a G&S ordered compared to lower limb procedures (incidence rate ratio (IRR)=0.2, p=0.009), but conversely, humerus/shoulder procedures were associated with a significantly higher rate of testing (IRR=3.0, p=0.037). Patients with higher ASA grades were found to have a significantly higher rate of testing compared to lower ASA grades (p=0.024). Additionally, female patients had approximately 25% fewer tests compared to males (IRR=0.75, p=0.04). Other factors like age, preoperative hemoglobin, and anemia were not significantly associated with the rate of G&S testing. A breakdown of the Poisson regression modelling results is shown in Table 4.

**Table 4:** Poisson regression modelling for multivariable analysis To assess independent predictors of the rate of G&S testing after adjusting for confounding variables. A p-value of <0.05 was considered statistically significant. IRR, incidence rate ratio; ASA, American Society of Anaesthesiologists

Variable	IRR	p-value
Sex (female)	0.75	0.04
Age (per year)	1	0.59
Limb (upper)	0.2	0.009
ASA grade	Trend	0.024
Preoperative hemoglobin	0.99	0.13
Fracture: humerus/shoulder	3	0.037
Anemia (yes)	1.22	0.33

## Discussion

The primary finding of zero postoperative red cell transfusions provides compelling evidence that routine preoperative G&S testing is not clinically justified for most patients undergoing ambulatory peripheral limb orthopedic trauma surgery. This 0% transfusion rate strongly supports the argument that there is no clinical utility of routine G&S testing in this specific low-risk cohort and only carries significant associated costs and inefficiencies. This aligns with low transfusion rates observed in other low-blood-loss surgical fields and provides specific data to inform orthopedic trauma practice. Results in this study are noteworthy due to the limited literature on the topic [11], with previous work almost exclusively focused on elective arthroplasty, which also indicates G&S testing is unnecessary due to low transfusion rates [14,15].

Despite the low objective risk, G&S samples were ordered in 34.3% of patients, resulting in 315 unnecessary tests. Our statistical analysis highlights that current practice is not driven by objective hematological markers (such as preoperative hemoglobin or age), but rather by factors that reflect the clinician's perceived risk profile. The significant association between G&S ordering and higher ASA grade suggests clinicians are primarily concerned with a patient's pre-existing physiological reserve and comorbidities, which may limit their ability to tolerate even minor blood loss. These findings may be related to previous analyses and reviews that highlight perioperative factors that increase risk of transfusion, including female sex, ASA grade ≥3, and preoperative anemia [16,17]. Similarly, the increased testing rate for proximal structures such as the tibia and humerus/shoulder, compared with distal sites, suggests a procedural complexity bias and aligns with previous studies on their blood loss and potential need for transfusion [8,18,19]. This indicates that G&S testing is currently a risk-aversion default rather than a targeted intervention.

While the risk of a catastrophic, life-threatening hemorrhage remains a rare possibility, a system of routine testing for all patients is an inefficient way to mitigate this risk. Our data strongly advocate for the immediate discontinuation of routine G&S testing for this cohort. We recommend the implementation of a selective, risk-stratification protocol. This protocol could consider limiting G&S testing only to patients meeting specific criteria, such as those with: (1) an ASA grade ≥3, (2) a procedure involving segments proximal to the elbow or knee, or (3) a preoperative hemoglobin level below the current institutional transfusion trigger. Furthermore, safety can be maintained by ensuring optimal intraoperative hemostatic strategies such as tourniquet use, diathermy, and antifibrinolytic agents, along with the immediate availability of group O Rh-negative packed red cells in or near the operating theater, as suggested in other studies [20-22]. This would provide a universally compatible blood product if an emergency transfusion were required before full cross-matching.

The 315 unnecessary G&S samples processed in this 12-month period represent a significant financial burden. At a conservative institutional cost of £7.29 per test, the cost in this single NHS trust was approximately £2,296. Extrapolated nationally and across multiple trusts, the cumulative wasted expenditure could be substantial, with other trusts documenting a significantly higher G&S cost and therefore cumulative load [11,23]. Moreover, this wasted resource has a direct environmental impact. Given that each blood sample process is estimated to generate 0.43 kg of CO_2_ equivalent [24], the unnecessary testing contributed approximately 135.5 kg of excess CO_2_ in a single year. By implementing a risk-stratified protocol, orthopedic units can demonstrate effective resource stewardship and directly contribute to the NHS's broader environmental sustainability goals.

The primary limitations of this study include its single-center, retrospective design, which may limit generalizability and introduce selection bias related to the definition of "ambulatory" procedure. The study population was strictly defined to include only peripheral limb trauma, meaning the findings cannot be extrapolated to higher-risk procedures (e.g., hip/femoral fractures). Additionally, the reliance on documented G&S orders may not fully account for all possible drivers of clinician decision-making. Despite these limitations, the robust 0% postoperative transfusion rate across a well-defined cohort provides a strong evidence base for policy change.

## Conclusions

This study provides compelling evidence that routine preoperative G&S testing is unnecessary for patients undergoing ambulatory peripheral limb orthopedic trauma surgery due to the absence of postoperative red cell transfusion. Current practice is poorly correlated with objective risk factors and contributes to a significant, quantifiable economic and environmental burden. We advocate for the discontinuation of routine G&S testing in this patient cohort and recommend the development of a mandatory, evidence-based risk-stratification protocol to improve resource utilization, reduce costs, and support the environmental sustainability of orthopedic practice.
